# Optical changes in THP-1 macrophage metabolism in response to pro- and anti-inflammatory stimuli reported by label-free two-photon imaging

**DOI:** 10.1117/1.JBO.25.1.014512

**Published:** 2020-01-17

**Authors:** Isabel S. Smokelin, Craig Mizzoni, Josh Erndt-Marino, David L. Kaplan, Irene Georgakoudi

**Affiliations:** a Tufts University, Department of Biomedical Engineering, Medford, Massachusetts, United States; b Tufts University, Sackler School of Graduate Biomedical Sciences, Cell, Molecular, and Developmental Biology Program, Boston, Massachusetts, United States

**Keywords:** macrophages, metabolism, two-photon excited fluorescence, NAD(P)H, inflammation, mitochondria

## Abstract

Temporal changes in macrophage metabolism are likely crucial to their role in inflammatory diseases. Label-free two-photon excited fluorescence (TPEF) and fluorescence lifetime imaging microscopy are well suited to track dynamic changes in macrophage metabolism. We performed TPEF imaging of human macrophages following either pro- or an anti-inflammatory stimulation. Two endogenous fluorophores, NAD(P)H and FAD, coenzymes involved in key metabolic pathways, provided contrast. We used the corresponding intensity images to determine the optical redox ratio of FAD to FAD + NAD(P)H. We also analyzed the intensity fluctuation patterns within NAD(P)H TPEF images to determine mitochondrial clustering patterns. Finally, we acquired NAD(P)H TPEF lifetime images to assess the relative levels of bound NAD(P)H. Our studies indicate that the redox ratio increases, whereas mitochondrial clustering decreases in response to both pro- and anti-inflammatory stimuli; however, these changes are enhanced in pro-inflammatory macrophages. Interestingly, we did not detect any significant changes in the corresponding NAD(P)H bound fraction. A combination of optical metabolic metrics could be used to classify pro- and anti-inflammatory macrophages with high accuracy. Contributions from alterations in different metabolic pathways may explain our findings, which highlight the potential of label-free two-photon imaging to assess nondestructively macrophage functional state.

## 1 Introduction

Macrophages are a diverse group of myeloid cells that are involved in a wide range of immune system responses.^1^ They play a key role in the progression of several diseases related to inflammation, such as Parkinson’s disease, type 1 diabetes, and rheumatoid arthritis.^2^ In the absence of a stimulus, macrophages exist in a “resting” state (M0) but can be induced to enter different phenotypes, depending on the nature of the environmental stimuli or the stage of the response. Following injury or infection, macrophages can take on a “classic” pro-inflammatory activation state and phagocytize invading pathogens, cellular debris, and apoptotic cells.^3^^,^^4^ They also produce high levels of pro-inflammatory cytokines such as interleukin-1 beta (Il-1β), Il-6, Il-12, and tumor necrosis factor alpha (TNF-α)^3^ and low levels of Il-10.^1^^,^^5^ In the wound healing process, the anti-inflammatory macrophage phenotype dominates during later stages of repair.^4^ These macrophages release anti-inflammatory cytokines, such as Il-10, CCL18, CCL22,^3^^,^^5^ and Il1-receptor agonist (IL-1-ra),^6^^,^^7^ and aid with the repair of damaged tissue.^1^ Anti-inflammatory macrophages also stimulate extracellular matrix production and angiogenesis.^4^ Both pro-inflammatory and anti-inflammatory macrophage phenotypes are essential to the inflammatory response and the wound-healing process. However, macrophages often exhibit both “pro-inflammatory” and “anti-inflammatory” markers simultaneously and may take on a phenotype somewhere between both ends of the spectrum depending upon relative cytokine levels created by the tissue environment or stimuli.^8^ Thus understanding their function at any particular time point or within a particular tissue location is very challenging using traditional staining and/or biochemical assays.

A number of studies, performed primarily with murine macrophages, have identified significant changes in the relative utilization of glycolysis, oxidative phosphorylation, glutaminolysis, and fatty acid oxidation (FAO).^7^^,^^9^ However, studies performed with human macrophages suggest that distinct metabolic changes may be associated with pro- and anti-inflammatory macrophages from those observed with murine macrophages. For example, murine macrophages switch from oxidative phosphorylation to glycolysis for energy production in response to inflammatory stimuli, whereas human macrophages continue to rely on oxidative phosphorylation as a major energy source.^10^ Such findings further highlight the complexity of understanding macrophage function and the dependence on origin and local environment.

Label-free, two photon imaging has been shown in numerous studies to be a highly sensitive, nondestructive tool for assessing cellular metabolic function not only in cell cultures but also in three-dimensional tissues, both *in vitro* and *in vivo*.^11^^–^^17^ In fact, we have recently shown that a combination of optical metabolic readouts extracted from analysis of intensity and lifetime two-photon excited fluorescence (TPEF) images based on signal from NAD(P)H and FAD are sensitive to changes in the main metabolic pathways that have been reported to be altered with changes in macrophage functional state.^7^^,^^9^^,^^18^ NAD(P)H and FAD are coenzymes that fluoresce naturally and are involved in many metabolic pathways.^18^ Thus in this study, we aimed to determine whether multiparametric, label-free, two-photon imaging can be employed to provide insights regarding macrophage activation.

We assessed pro-inflammatory macrophage responses induced by lipopolysaccharide (LPS), and anti-inflammatory responses elicited by interleukin-4 (IL-4), following established procedures.^6^^,^^8^^,^^19^^,^^20^ Therefore, we refer to classically activated, pro-inflammatory macrophages as M[LPS] and alternatively activated, anti-inflammatory macrophages as M[IL-4]. As in our previous studies, we used intensity TPEF NAD(P)H and FAD images to extract the optical redox ratio, defined as FAD/[NAD(P)H + FAD]^18^^,^^21^^,^^22^ and further analyzed the intensity fluctuation patterns within intensity NAD(P)H TPEF images to extract a quantitative metric of mitochondrial clustering or fragmentation.^23^^–^^26^ We also acquired NAD(P)H TPEF lifetime [fluorescence lifetime imaging microscopy (FLIM)] images and analyzed them using the phasor approach in Fourier space to determine NAD(P)H bound fraction.^18^^,^^27^^,^^28^ Finally, we evaluated the combination of optical metabolic changes we observed and their potential to report macrophage functional state.

## 2 Materials and Methods

### 2.1 Cell Culture and Differentiation of THP-1 Monocytes

THP-1 human monocytes (ATCC; TIB-202™) were grown in suspension culture through addition of culture media into the flask, keeping the density within $3\times 10^{5}$ to $1\times 10^{6}\text{ cells}/\mathrm{mL}$. Culture media for all experiments was RPMI 1640 containing L-glutamine (Gibco) supplemented with 10% fetal bovine serum (Gibco) and 1% antibiotic solution (10,000  IU/mL penicillin, 10,000  μg/mL streptomycin, Gibco). To induce differentiation toward a macrophage phenotype, THP-1 cells were seeded in a 24-well glass-bottom plate at a concentration of $∼2\times 10^{5}\text{ cells}/\mathrm{mL}$ using 1 mL of cell suspension in culture media containing 10  ng/mL Phorbol 12-myristate 13-acetate (PMA; Fisher Ca. 12-011). Low-dose PMA differentiation was chosen because it has been shown to be more representative of human macrophage responses, may allow for enhanced responsiveness to weaker stimuli, and has been utilized to investigate human macrophage metabolism with THP-1 cells.^29^^–^^33^ After 3 days, PMA differentiated monocytes were further driven toward different cell states through replacement of the PMA media with 1 mL of culture media alone (control) or culture media supplemented with either 100  ng/mL LPS (salmonella enterica, Sigma) or 20  ng/mL human recombinant IL-4 (R&D Systems). Even though THP-1 monocytes are derived from a leukemia patient, PMA treated THP-1 cells are an established model for human monocyte-macrophage differentiation.^29^^–^^32^^,^^34^ They have been shown to mimic native macrophages with regards to morphology, secretory products, membrane receptors, and antigens, and to be superior in that regard compared to other human myeloid cell lines such as HL-60, KG-1, U937, and HEL cell lines. For these reasons, we selected them as a model for this study.^34^

### 2.2 Assessment of Macrophage Phenotype

Media was collected from M[0], M[LPS], and M[IL-4] wells after 24 h of culture and centrifugation at 10,000g for 5 min. Levels of TNF-α and interleukin (IL) -6 and -10 were measured from culture media samples using a human magnetic bead analyte kit (EMD Millipore) and the MAGPIX detection system (Luminex) according to manufacturer’s protocols and as previously reported elsewhere.^35^ Measurements were performed from at least three independent wells from each treatment group.

### 2.3 Assessment of Mitochondrial Polarization

Mitochondrial membrane potential ($V_{\text{mito}}$) assessment was performed using a fluorescent lipophilic cationic dye [tetramethylrhodamine methyl (TMRM), ThermoFisher]. As a cationic dye, TMRM is sequestered in a complex fashion into the cell cytosol and mitochondrial membrane, with increasing accumulation and fluorescence intensity corresponding to increasing charge, or hyperpolarization, of the mitochondria. PMA differentiated THP-1 macrophages were lifted with TrypLE Express (Gibco) for 5 min at 37°C and pelleted with 130g for 5 min. THP-1 pellets were resuspended to achieve a concentration of 0.5 to $1.0\times 10^{6}\text{ cells}/\mathrm{mL}$ in tyrode’s solution containing 20 nM TMRM and 3  μL/mL 7-aminoactinomycin D (7-AAD; Abcam) and incubated at 37°C for 40 min. The labeled cells were analyzed using a flow cytometer (FACSCalibur, BD Bioscience) with 488 nm excitation and the FL-2 (585±21  nm for TMRM) and FL-3 (650 nm longpass for 7-AAD) emission filters. After gating for forward scatter versus side scatter and viability, we analyzed the median fluorescence intensity (MFI) values of TMRM. Assuming: (1) operation in nonquench mode of the dye, (2) constant plasma membrane voltage, (3) loading of the dye in equilibrium, (4) equivalent mitochondrial volume fractions, and (5) equivalent apparent activity coefficient ratios, lower intensity values are indicative of mitochondrial depolarization, whereas higher MFI values indicate mitochondrial hyperpolarization.^36^^–^^39^ Measurements were performed on five independent samples.

### 2.4 Image Acquisition

Images of M[0], M[LPS], and M[IL-4] macrophages were acquired using a Leica TCS SP8 microscope with a 40×, 1.1 NA water immersion objective 6 and 24 h after the addition of control, LPS-, or IL-4-containing media. NAD(P)H signal was collected at 755 nm excitation/460±20  nm emission and FAD was detected at 860 nm excitation/525±25  nm emission. At both time points, at least four fields of view were examined per well, and at least two wells were examined per cell type. This entire experiment was repeated three independent times. For the intensity images, 1024×1024  pixel frames were acquired at a speed of 1  s/frame and a zoom factor of 0.75, yielding a pixel size of 0.379  μm and theoretical lateral resolution of 0.412  μm at 755 nm. Eight frames per field were averaged for each intensity image. The illumination power at the sample for both intensity and lifetime imaging was ∼10  mW at 755 nm and ∼14  mW at 860 nm, but day-to-day variations were taken into account during image preprocessing. Image size, frame rate, speed, and zoom factor were kept constant across all experiments. Following the intensity-based imaging, 512×512  pixel NAD(P)H fluorescence lifetime microscopic images were acquired using the same excitation/emission settings as for the intensity images and a PicoHarp 300 time-correlated single-photon counter (integrated in the Leica microscope), with a 200-ps resolution. The integration time for each frame was 60 s. HyD detectors were used for the acquisition of all images.

### 2.5 Image Analysis

#### 2.5.1 Optical redox ratio assessment

We normalized images based on illumination power used per experiment and co-registered the NAD(P)H and FAD images using cross-correlation algorithms.^21^^,^^28^ Nuclei and background fluorescence were excluded using a binary mask with Otsu’s threshold method to minimize noise unrelated to metabolism.^28^ We then calculated pixel-wise the optical redox ratio as FAD/[FAD + NAD(P)H], using the fluorescence intensity of NAD(P)H and FAD TPEF images.

#### 2.5.2 Mitochondrial clustering analysis

Mitochondrial clustering was evaluated based on analysis of the TPEF NAD(P)H intensity fluctuations using methods we described in detail previously.^18^^,^^23^^–^^26^ The approach relies on the fact that the intensity of NAD(P)H fluorescence is enhanced by about 10-fold when bound in the mitochondria.^40^ We have shown that the frequency patterns of the NAD(P)H intensity fluctuations can be characterized to yield metrics of mitochondrial networking and fragmentation, and validated the sensitivity of this approach to mitochondrial organization changes in several studies performed with similar resolution capabilities.^18^^,^^23^^–^^26^ Briefly, a binary mask was made by excluding low-intensity regions of the images (corresponding to the background and weakly fluorescent nuclei), leaving behind only the cytoplasmic areas of the cell. Then the mitochondrial signal in the cell cytoplasm was cloned and repositioned randomly in the dark pixels to reduce the impact of the dominant nuclear and cell border features in the analysis.^23^ Next, the power spectral density (PSD) of each frame was calculated based on the squared amplitude of its 2-D Fourier transform. The PSD was fit with a power law ($∼\alpha k^{\beta }$) within spatial frequencies (k) in the 0.12- to $1.2\text{-}\mu m^{-1}$ range, corresponding to size features from 0.833 to 8.33  μm (a range that targets the approximate size of mitochondria, which tend to be ∼1 to 6  μm in length in murine macrophages and 700 nm wide and between 1 to 10  μm in length in fibroblasts).^41^^,^^42^ The absolute value of the slope of this linear fit represented the mitochondrial clustering value β. Since clone stamping is a randomized process, it was performed five times per image, with the mean β value for mitochondrial clustering across all trials used for final reporting. Higher β values are indicative of fragmented mitochondrial networks, whereas lower β values are indicative of more fused mitochondrial networks.^18^^,^^23^^–^^26^

#### 2.5.3 NAD(P)H bound fraction

The NAD(P)H bound fraction was determined based on analysis of the fluorescence intensity decay profiles acquired using FLIM. The phasor approach^27^^,^^43^ was used to assess the NAD(P)H bound fraction as described previously.^18^ Briefly, each fluorescence decay profile was transformed to Fourier space, and the real and imaginary parts of the Fourier transform were used as the x and y coordinates in phasor space, respectively. Data from a typical image resulted in a collection of points that formed an ellipsoid shape. A line fit to the phasor ellipsoid crossed the universal semicircle at locations that corresponded to the short (τ1) and long (τ2) lifetime of NAD(P)H. The NAD(P)H bound fraction was estimated for each pixel by projecting its phasor onto the line and considering its relative location with respect to the short and long lifetimes. Color-coded maps were then created using MATLAB to visualize bound fraction at each pixel.^18^

### 2.6 Statistical Analysis

Statistical analysis was completed with SPSS software, and p values<0.05 were considered significant and were further classified into three ranges from 0.01 to 0.05, 0.001 to 0.01, and <0.001. All metrics were first normalized to the experiment’s 6-h control average to account for interexperiment variations. For redox ratio, NAD(P)H bound fraction and β clustering, a two-factor ANOVA followed by a Tukey *post hoc* test with time and treatment as the two independent variables, and redox ratio, bound fraction, and β value as the respective-dependent variables was performed. After, separate one-way ANOVAs each followed by a Tukey *post hoc* test to examine differences between individual variables were performed. For the TMRM data, a one-way ANOVA with treatment as the dependent variable was performed, followed by a Tukey *post hoc* test. For all two-factor and one-way ANOVAs, the assumption of homogeneity of variances was confirmed with Levene’s test. Canonical linear discriminant analysis was performed to assess the potential of the combined use of the three optical metabolic metrics to separate the different groups of macrophages for the 6-h and 24-h time points. The linear discriminant functions were established and leave-one-out cross validation was performed to assess the classification accuracy of the models using SPSS. Normality and independence criteria were met and all data points were valid for this analysis. Through the course of the study, 18 independent samples (3 experiments × 3 treatment groups per experiment × 2 time points) were processed and each macrophage treatment group had 24 observations (3 experiments × 2 wells per group × 4 fields per well) at both the 6- and 24-h time point.

## 3 Results

**Fig. 1:**
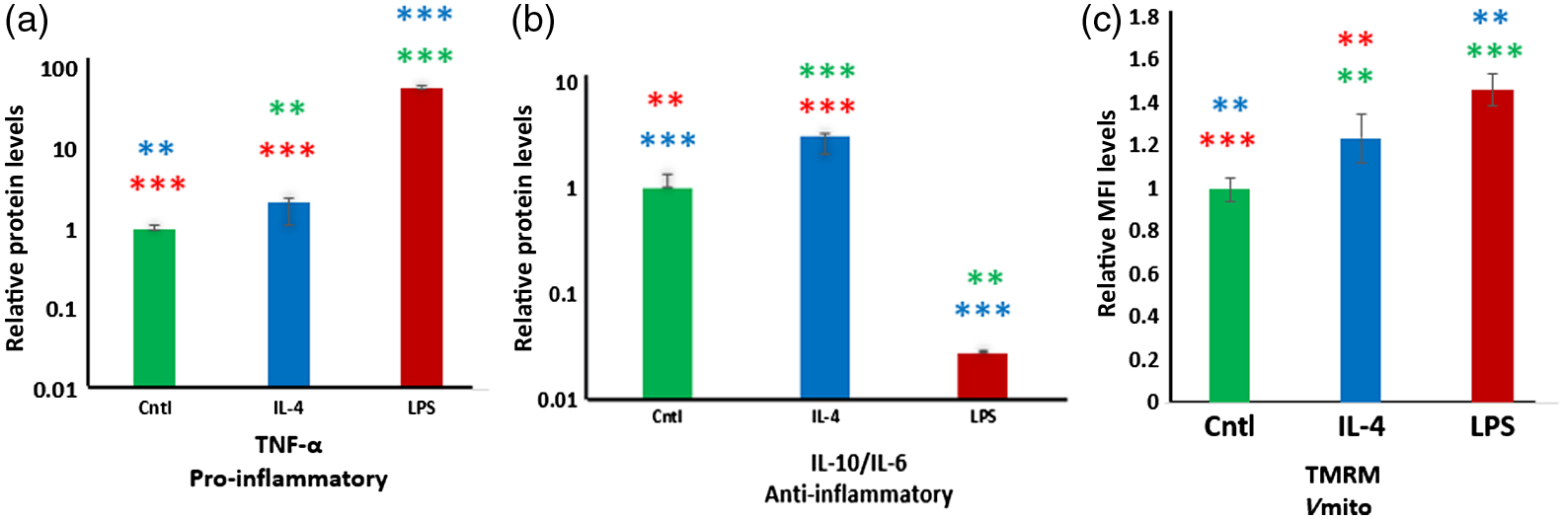
Phenotypic changes occurring in control, M[Il-4] and M[LPS]. Relative protein levels of pro-inflammatory (a) and anti- inflammatory (b) cytokines secreted into the media over 24 hours of culture. Note: *(green), *(blue) denote statistically significant from control and M[IL-4], respectively. (c) Median fluorescence intensity (MFI) for control, M[Il-4] and M[LPS] after 24 hours. Note: *(green), *(blue) denote statistical significance from control and IL-4, respectively. One, two and three significance symbols correspond to p-values from 0.01 to 0.05, 0.001 to 0.01, and <0.001, respectively.

To confirm that the pro- and anti-inflammatory stimulation protocols had the desired functional effects on the macrophages, we assessed the levels of cytokines that are characteristic for each type of response. M[LPS] exhibited a significant increase in the level of pro-inflammatory cytokine TNF-α [Fig. 1(a)] and a significant decrease in the ratio of anti-inflammatory cytokines IL-10/IL-6 [Fig. 1(b)] relative to M[0] and M[Il4] controls, confirming the acquisition of a pro-inflammatory phenotype. The acquisition of an anti-inflammatory phenotype in M[IL-4] was confirmed by an increase in the levels of anti- to pro-inflammatory cytokines, IL-10/Il-6 [Fig. 1(b)].^1^^,^^3^^,^^5^ A ratio was used to represent data in a format that is widely used when analyzing macrophage phenotype.^44^ TMRM staining (indicated by MFI), an indicator mitochondrial membrane potential, was also measured at the 24-h time point. The MFI of M[LPS] was significantly higher than that of both M[0] (p<0.0001) and M[IL-4] (p=0.002), and the MFI of M[Il4] was significantly higher than that of M[0] (p=0.0033) [Fig. 1(c)]. These results indicated that both pro- and anti-inflammatory stimuli led to a higher mitochondrial potential (i.e., hyperpolarization) relative to the control group.

**Fig. 2:**
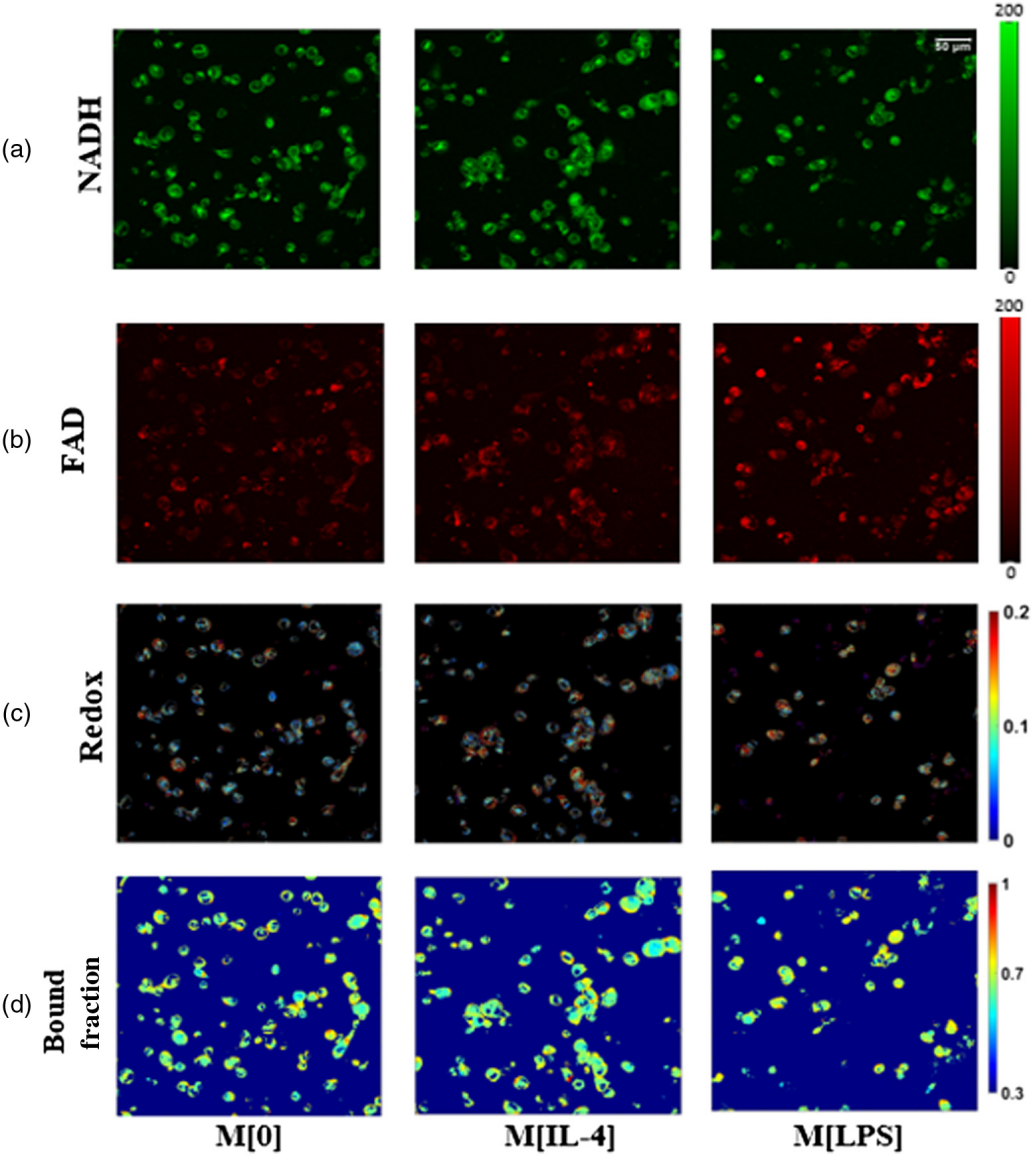
Representative M[0], M[Il4], and M[LPS] images of (a) NAD(P)H and (b) FAD endogenous fluorescence at the 24-h time point with the respective (c) redox ratio map and (d) NAD(P)H bound fraction map for each. Scale bar=50  μm.

**Fig. 3:**
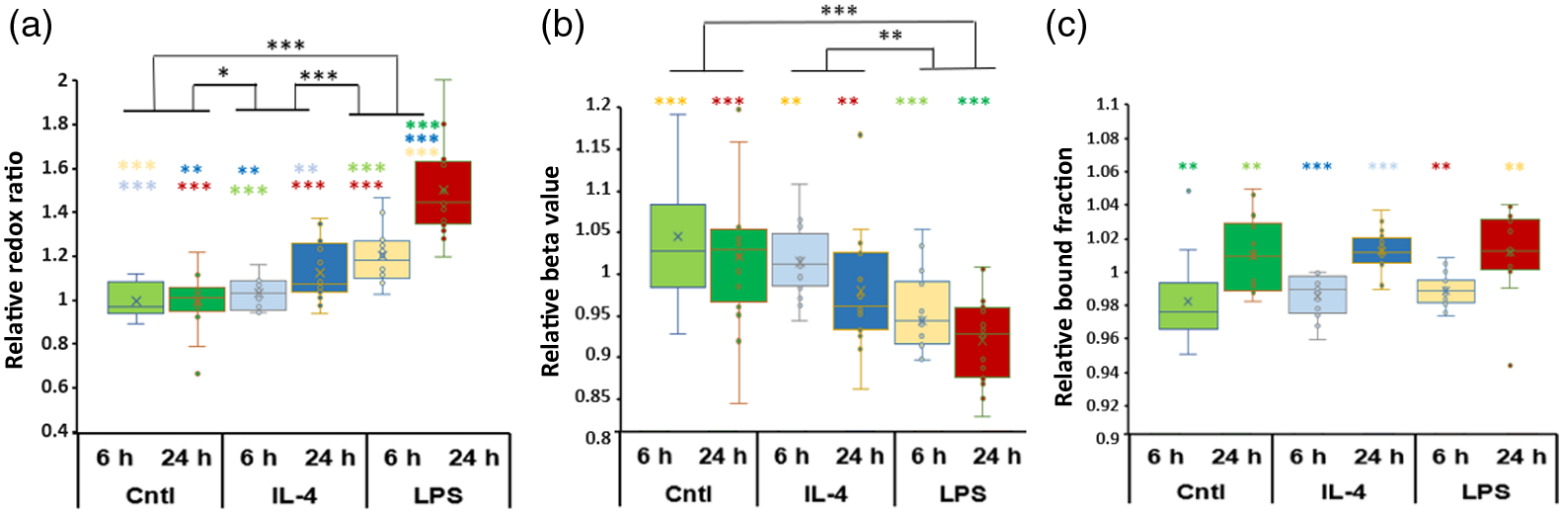
Relative (a) optical redox ratio, (b) mitochondrial clustering, and (c) NAD(P)H bound fraction values for control, M[IL-4], and M[LPS] groups following 6 and 24 h of treatment. One, two, and three significance symbols correspond to p values from 0.01 to 0.05, 0.001 to 0.01, and <0.001, respectively. The colored * represents the significant difference of the group to the corresponding color’s condition. Eight fields of view from two different wells were imaged per treatment group at each time point, and the experiment was replicated three times.

M[0], M[IL-4], and M[LPS] were imaged at a 6- and 24-h time point using TPEF and FLIM and representative images are shown for the 24-h time point in Fig. 2. Redox ratio coded [from the combination of FAD and NAD(P)H intensity images] and bound-fraction coded (from analysis of the FLIM images) images are also shown. Corresponding phasor plots are included in Fig. S1 in the Supplementary Material. Results from quantitative analysis of all the images acquired are shown in Fig. 3. Relative to M[0] (control macrophages), there was a significant increase in the redox ratio in both M[IL-4] (p=0.014) and M[LPS] (p<0.001) groups. M[LPS] redox ratio was also significantly higher than that of M[IL-4] (p<0.001) [Fig. 3(a)]. No significant differences were detected between 6 and 24 h for the control group, whereas such differences were detected for both M[IL-4] (p<0.01) and M[LPS] (p<0.001) treatments. The M[LPS] mitochondrial clustering values decreased significantly relative to both M[0] (p<0.001) and M[IL-4] (p=0.0011) [Fig. 3(b)]. These p values report differences across experimental groups, irrespective of time. Interestingly, we did not detect any significant changes between treatment groups for the NAD(P)H bound fraction, even though significant differences were detected for all groups between the images acquired at 6 and 24 h [Fig. 3(c)]. The corresponding short and long lifetimes assessed based on the intercept locations of the phasor principal axis along the universal semicircle also did not exhibit any significant differences between groups. For example, at 24 h $\tau_{1}$ values were equal to 0.31±0.004, 0.31±0.002, and 0.31±0.003 and $\tau_{2}$ values were 4.1±0.26, 3.97±0.09, and 3.95±0.22 for the control, M[IL-4], and M[LPS] groups, respectively.

Five supplemental videos (Videos S1–S5) listed in the Appendix (Section 6) enable multiple angle visualization of the changes in optical redox ratio, NAD(P)H bound fraction, and mitochondrial clustering across macrophage treatment groups at 6- and 24-h. Each ellipsoid encompasses 75% of data coverage. Videos S1 and S2 provide comparisons of the metabolic readouts among the different groups of macrophages (M[0], M[IL-4], and M[LPS]) at either 6 h (Video S1) or 24 h (Video S2) following the onset of activation. Videos S3–S5 provide comparisons of the metabolic readouts for each group of macrophages (M[0]-Video S3, M[IL-4]-Video S4, M[LPS]-Video S5) at 6-h and 24-h. Each ellipsoid encompasses 75% of data coverage. When the relative changes in the three optical metrics are used as a potential means to classify macrophages into M[0], M[IL-4], and M[LPS] groups, we achieve an original classification accuracy of 75.3%, and cross-validated classification accuracy of 71.1% at the 6-h time point, and an original classification accuracy of 85.4% and cross-validated classification accuracy of 83.3% at the 24-h time point. These numbers confirm the higher level of changes detected following the longer stimulation time point. In fact, we note that the cross-validated accuracy for distinguishing only the LPS-activated macrophages from controls following 24 h of activation is 93.8%. These levels of accuracy suggest that ultimately a combination of these metrics may be useful in assigning a probability that a macrophage is more or less likely to be in a pro- or anti-inflammatory functional state without the need for an exogenous label.

**Fig. 4:**
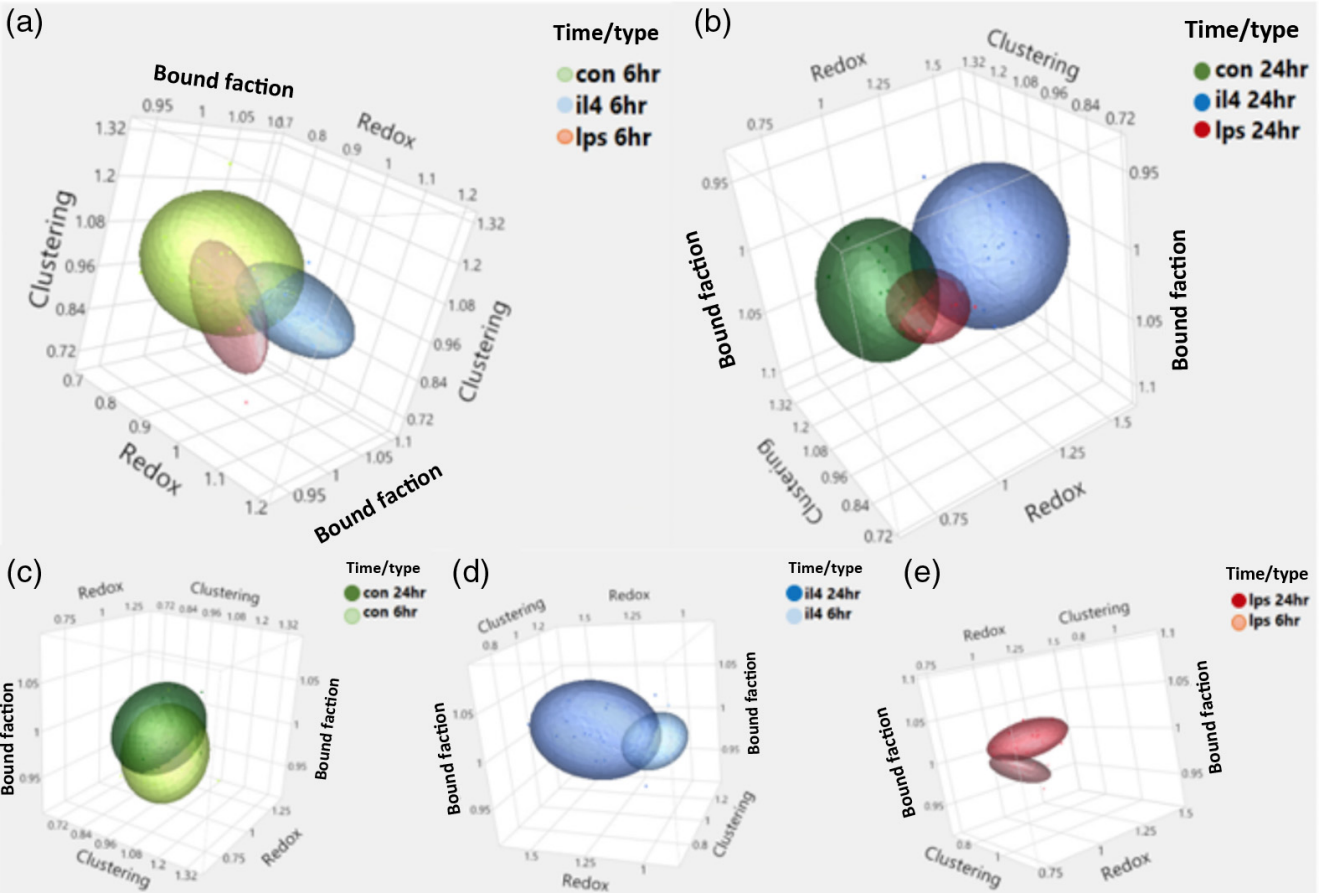
Visualization of the relative changes in redox ratio, bound fraction, and mitochondrial clustering in M[0], M[Il4], and M[LPS] at the (a) 6-h and (b) 24-h time points. The colors used for treatment group are indicated in the top right of each plot and are consistent across all five graphs. Plots (c) M[0], (d) M[IL-4], and (e) M[LPS] represent the relative changes in the three optical metrics within each treatment group across the 6-h and 24-h time point. For all plots, the ellipsoid represents 75% of data coverage.

## 4 Discussion

In this study, we sought to determine the potential of label-free, two-photon imaging to report functional metabolic differences between human macrophages with either a pro- (M[LPS]) or an anti-inflammatory (M[IL-4]) phenotype. We used label-free, intensity and lifetime TPEF to assess quantitatively the signal emanating from NAD(P)H and FAD and extracted complementary metabolic metrics, including the optical redox ratio, defined as FAD/[NAD(P)H + FAD], mitochondrial clustering, and the NAD(P)H bound fraction. We chose to examine these three optical metrics together, as we have shown recently that such a multiparametric approach can provide more specific insights regarding the nature of metabolic perturbation that yields changes in optical metabolic readouts.^18^ For example, simultaneous decreases in optical redox ratio and NAD(P)H bound fraction are indicative of both enhanced glycolytic metabolism and unsaturated FAO, but mitochondrial clustering increases with the former, whereas it remains constant with the latter.^18^

In these studies, we observe significant changes in redox ratio and mitochondrial clustering in M[IL-4] and M[LPS] in response to pro- and anti-inflammatory stimuli. The trends for both cell types are the same, but the level of change for each metric is distinct depending on the nature of the inflammatory stimulus. Interestingly, no net significant changes are observed between resting/control M[0] and either M[LPS] or M[IL-4] macrophages with regards to their fluorescence lifetime characteristics, as reported by the NAD(P)H bound fraction. Linear discriminant analysis based on the combination of optical metabolic parameters we extract and leave-one-out cross-validation yield classification accuracies for M[0], M[IL-4], and M[LPS] macrophages of 83.3% following 24 h of treatment. Thus our results suggest that we can detect metabolic changes associated with distinct macrophage functional states; however, these metabolic differences are subtle and need further studies to be fully exploited to improve our understanding of this important cell type in the context of inflammation.

The observed increases in optical redox ratio and decreases in mitochondrial clustering are consistent with enhanced levels of oxidative phosphorylation and glutaminolysis.^18^ Both of these processes utilize mitochondrial NAD(P)H, leading to its depletion, and thus a higher optical redox ratio (Fig. S2 in the Supplementary Material). Such changes in the optical redox ratio have been validated with mass spectrometry measurements and biochemical assays and have been reported for engineered epithelial tissues and primary human epithelial cells.^18^^,^^22^ Further, fused mitochondrial networks are known to form as cells switch from glycolysis to oxidative phosphorylation in human skin epithelia and have been reported with corresponding decreases of mitochondrial clustering assessments based on similar analysis of NAD(P)H TPEF images.^26^ Mitochondrial clustering decreases in response to glutaminolysis, yielding more fused networks to help maintain ATP levels, have also been reported from equivalent assessments of engineered epithelial tissues and human primary cell cultures.^18^^,^^22^

Our optical redox ratio and mitochondrial clustering results are inconsistent for M[LPS] in studies of murine macrophages, but consistent with studies performed with human M[LPS]. In murine macrophages, it is established that in response to pro-inflammatory stimuli glycolysis is enhanced while oxidative phosphorylation (oxphos) is limited due to a dysfunctional tricarboxylic acid (TCA) cycle.^45^^,^^46^ A significant decrease in the NAD(P)H bound fraction as assessed by corresponding lifetime measurements has been reported in LPS activation of murine macrophages and is consistent with enhanced glycolysis.^47^ However, recent studies suggest that proinflammatory human peripheral blood-derived macrophages rely on oxidative phosphorylation for energy production^10^ and do not produce nitric oxide or regulate reactive oxygen species production, thus contradicting the assumed reprogramming of the TCA cycle for glycolytic metabolism as is seen in their murine counterparts.^7^^,^^10^ In addition, human macrophage responses to β-glucan (an immunostimulant glucose polymer that induces some similar effects in macrophages as LPS)^48^ have been found to be contingent upon the glutaminolysis pathway,^49^ supporting a potential role for enhanced glutaminolysis under pro-inflammatory conditions, consistent with our findings.

The increase in optical redox ratio and decrease in β clustering for M[IL-4] is consistent with enhanced oxidative phosphorylation and glutaminolysis detected in murine macrophages.^9^^,^^50^^,^^51^ M[IL-4] produces ATP via oxidative phosphorylation linked to an intact, oxidative TCA cycle.^9^ The TCA cycle is thought to be fueled by both glutamine and fatty acids for energy production, rather than being repurposed to generate ROS, as is hypothesized in M[LPS].^9^^,^^52^ An increase in NAD(P)H bound fraction assessed from lifetime measurements has been reported in M[IL-4] murine macrophages and is also consistent with this type of metabolic perturbation.^47^ However, we note that in our studies with human macrophages, we do not detect any significant changes in NAD(P)H bound fraction values.

The role of FAO in M[LPS] and M[Il-4] in human macrophages is not clear. In FAO, fatty acids are converted to acetyl CoA, which then drives the TCA cycle to support oxidative phosphorylation^7^ (Fig. S2 in the Supplementary Material). For the past decade, studies using murine macrophages have indicated that FAO is required and essential for anti-inflammatory macrophage activation.^7^ However, recent studies have shown that inhibiting FAO in human macrophages does not inhibit Il-4-based gene expression, indicating that it may not be an essential pathway.^53^ Thus the exact role of FAO in anti-inflammatory macrophage activation, and how it may differ between murine and human systems is not clear.^7^ If FAO is enhanced in M[Il-4], then the combination of enhanced glutaminolysis, oxidative phosphorylation, and FAO at varying levels may explain why we see differences in the levels of changes in redox ratio and mitochondrial clustering between M[IL-4] and M[LPS]. During FAO, NAD(P)H is produced, so redox ratio decreases.^18^ It is possible that while glutaminolysis and oxidative phosphorylation are driving up the redox ratio, FAO is decreasing it, thus yielding a cumulative redox ratio in M[Il-4] that is significantly higher than the control group but also significantly lower than the LPS group [Fig. 3(a), Fig. S2 in the Supplementary Material]. Likewise, while both M[LPS] and M[Il4] groups are likely impacted by glutaminolysis (yielding a lower β value), the contribution of FAO in M[Il4] would favor maintaining the levels of β clustering, resulting in the observed overall higher value for M[IL-4] than M[LPS] but not in significant differences between M[IL-4] and controls [Fig. 3(b)].

Another metric of macrophage metabolic function is mitochondrial membrane potential ($V_{\text{mito}}$ or $\Delta \psi_{m}$). $\Delta \psi_{m}$ is characterized as an electrical and proton gradient that serves as an energy storage bank to drive the ATP synthase.^54^ $\Delta \psi_{m}$ is believed to be maintained by ATP generated through glycolysis for the purpose of preventing apoptosis.^55^ Classically activated macrophages are characterized by mitochondrial hyperpolarization,^7^^,^^55^ while little is known about $\Delta \psi_{m}$ in alternatively activated macrophages. The connection between mitochondrial polarization and the three optical metrics is complex. Mitochondrial hyperpolarization can be linked to inhibited oxidative phosphorylation and higher free NAD(P)H levels.^56^ Thus the lack of statistically significant differences in the detected NAD(P)H bound fraction values may be the outcome of competing contributions. For example, while enhanced levels of glutaminolysis are expected to lead to an increase in the NAD(P)H bound fraction values, hyperpolarization and FAO lead to a decrease. Differences in the levels of engagement of these pathways by murine and human macrophages may explain discrepancies in the NAD(P)H bound fraction changes between our study and previous ones.^47^ More detailed imaging studies in combination with biochemical and mass spectrometry assays are needed to enable us to tease out the importance and contributions of each metabolic pathway and their impact on NAD(P)H bound fraction across different macrophage types.

Despite remaining limitations, our studies highlight the potential of label-free, multiparametric metabolic imaging to not only serve as a sensitive means of detecting functional changes associated with macrophage activation but also to enable more biochemically meaningful interpretations of the origins of the detected changes. The combination of metrics extracted from multimodal, label-free imaging, which included quantitative phase microscopy (QPM), autofluorescence (AF), and Raman spectroscopy for identifying morphological and biochemical changes has been reported previously for murine macrophages treated with 1000  ng/mL LPS for 24 h. In that study, a combination of intensity and texture features from the QPM and AF images were used as morphological metrics while principal component analysis-derived parameters from the Raman spectra were used to sense biochemical changes. The accuracy of penalized logistic regression models to distinguish activated from resting macrophages using either morphological or biochemical metrics was in the 84%- to 87%-range, whereas a combination of both types of data yielded only a moderate enhancement (88%). Nevertheless, this study demonstrated that a combination of morphological and biochemical metrics yielded better separation between activated and resting cells when compared to inducible nitric oxide synthase levels assessed via immunohistochemistry. In addition, this study highlighted that the derived morphological metrics exhibited sensitivity to the LPS dose, whereas biochemical metrics did not. Although this study was performed with murine not human macrophages and the main results focused on a significantly higher LPS dose than the one we employed in our study (1000 versus 100  ng/mL), we note that several of the metrics that are described as morphology metrics, such as the AF intensity and AF texture, are sensitive to overall levels of NAD(P)H image intensity and texture, which are clearly related to metrics of redox ratio and mitochondrial clustering reported in our study. One of the key differences is that here the different parameters are reported in a manner that has been shown to be correlated with distinct metabolic functions. Thus interpretation of our results in the context of metabolic function is more straightforward. Finally, a key advantage of the methods we rely on for our studies is that they are readily translatable to *in vivo* and more broadly to human studies. Although penetration depth of label-free multiphoton imaging is limited to a few hundreds of microns, inflammatory cells have already been imaged in humans *in vivo* in response to fractionated picosecond laser treatment relying on endogenous [primarily NAD(P)H] fluorescence.^57^ The initial studies reported by us and others motivate studies that aim to assess macrophage behavior *in vivo*, under conditions that are more physiologically relevant and enable more meaningful understanding of the behavior and role of macrophages. They also provide a foundation for interpreting optical metabolic responses that may ultimately be observed *in vivo*.

## 5 Conclusion

Label-free imaging techniques such as TPEF and FLIM can be used to track metabolic changes in macrophages over time. The relative changes observed in redox ratio, mitochondrial clustering, and NAD(P)H bound fraction in response to pro- and anti-inflammatory stimuli are likely influenced by the complex interaction of different metabolic pathways including oxidative phosphorylation, glycolysis, glutaminolysis, and FAO. Although further research is necessary to fully understand the nuanced impacts of macrophage activation on optical metabolic read-outs, our studies demonstrate the potential of nondestructive, label-free, two-photon imaging to assess metabolic perturbations that can be used to identify functional differences in human macrophages exposed to pro- and anti-inflammatory stimuli. Thus this approach could serve as an important tool in understanding the role these cells play in inflammatory responses within three-dimensional tissues and ultimately *in vivo*, dynamically over time.

## 6 Appendix

The following supplemental videos are provided:

Video S1-Visualization of the relative change in redox ratio, bound fraction, and mitochondrial clustering in M[0], M[Il4], and M[LPS] at the 6-h time point. [URL: https://doi.org/10.1117/1.JBO.25.1.014512.1]
Video S2-Visualization of the relative change in redox ratio, bound fraction, and mitochondrial clustering in M[0], M[Il4], and M[LPS] at the 24-h time point. [URL: https://doi.org/10.1117/1.JBO.25.1.014512.2]
Video S3-Visualization of the relative change in redox ratio, bound fraction, and mitochondrial clustering in M[0] across the 6-h and 24-h time point. [URL: https://doi.org/10.1117/1.JBO.25.1.014512.3]
Video S4-Visualization of the relative change in redox ratio, bound fraction, and mitochondrial clustering in M[IL-4] across the 6-h and 24-h time point. [URL: https://doi.org/10.1117/1.JBO.25.1.014512.4]
Video S5-Visualization of the relative change in redox ratio, bound fraction, and mitochondrial clustering in M[LPS] across the 6-h and 24-h time point. [URL: https://doi.org/10.1117/1.JBO.25.1.014512.5]
