# Supplementary material for: Optical changes in THP-1 macrophage metabolism in response to pro- and anti-inflammatory stimuli reported by label-free two-photon imaging
Source: J Biomed Opt. 2020 Jan 17;25(1):014512. doi: 10.1117/1.JBO.25.1.014512 (PMC7008597; doi:10.1117/1.JBO.25.1.014512)
Supplement: Supplementary file 1 [file JBO_025_014512_SD001.pdf]

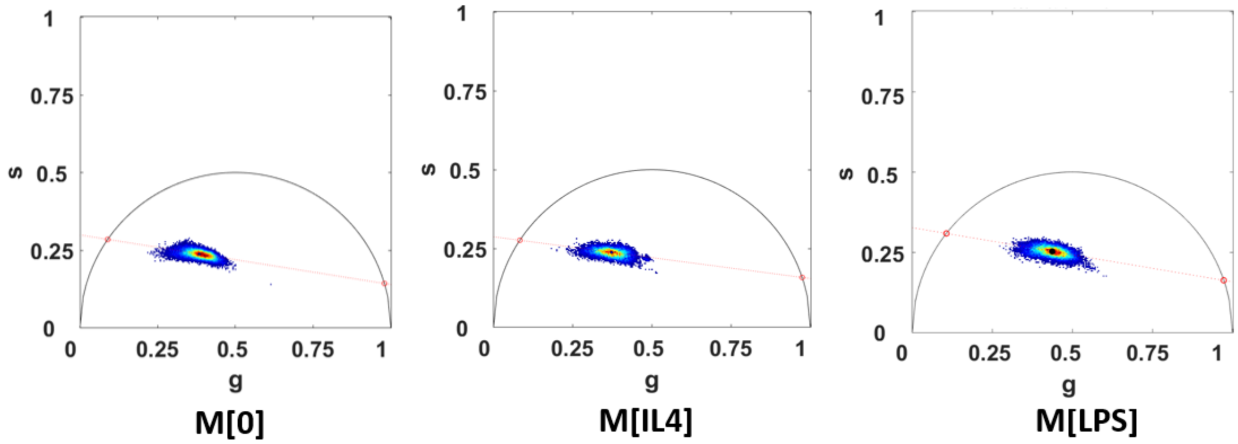

Fig. S1 Phasor plots of NAD(P)H lifetime for M[0], M[Il4] and M[LPS]. NAD(P)H was detected at 755nm excitation/460 ± 20nm emission and FAD was detected at 860 nm excitation/525 ± 25nm emission. Plots were created using MATLAB data processing algorithms.

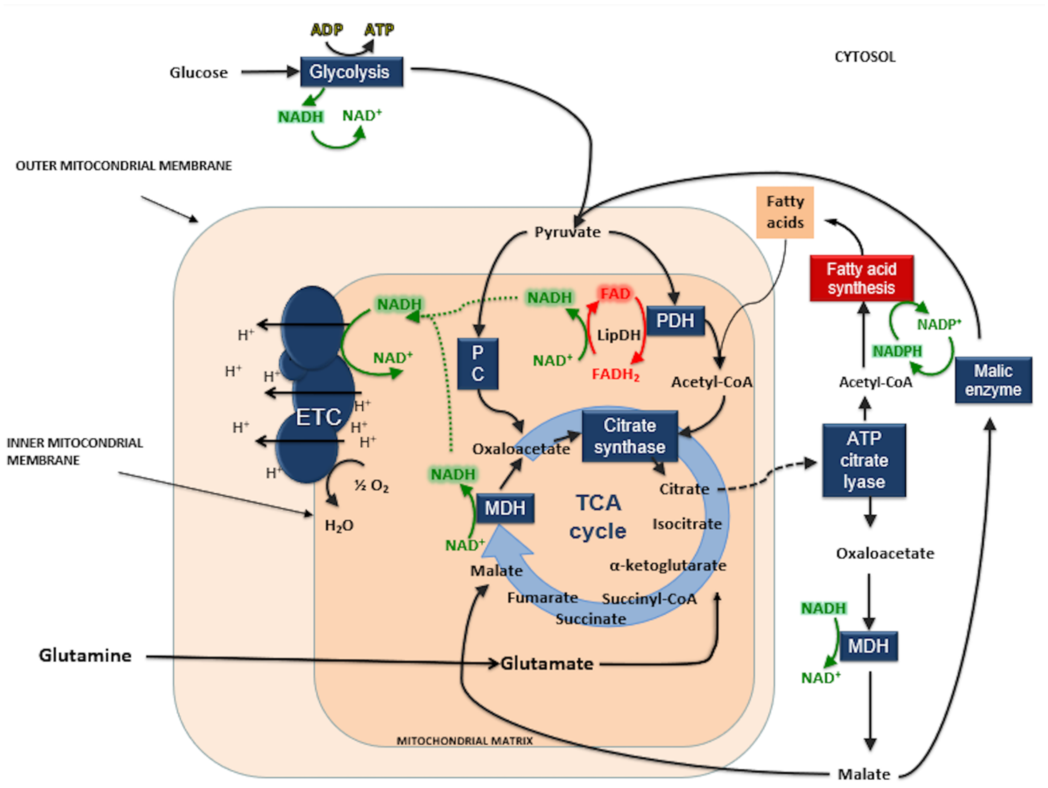

Fig. S2 Adaptation of schematic for key metabolic pathways involving NAD(P)H and FAD that may be relevant in macrophage activation (Liu, Zhiyi et al. 2018.)
